# Dental Care and Oral Health Within the Framework of Bulgarian Public Health Financing

**DOI:** 10.3390/healthcare13233055

**Published:** 2025-11-25

**Authors:** Ralitsa Raycheva, Ivelina Popova-Sotirova, Nina Musurlieva

**Affiliations:** Department of Social Medicine and Public Health, Faculty of Public Health, Medical University Plovdiv, 4002 Plovdiv, Bulgaria; ivelina.popova@mu-plovdiv.bg (I.P.-S.); nina.musurlieva@mu-plovdiv.bg (N.M.)

**Keywords:** dental care, oral health, health insurance, public financing, National Health Insurance Fund (NHIF), co-payments, healthcare accessibility, health inequalities, universal health coverage

## Abstract

**Background/Objectives**: Oral health is a critical component of public health, yet disparities in access and financing remain significant. In Bulgaria, dental services are financed through the National Health Insurance Fund and patient co-payments, with coverage differing between children and adults. The aim of this study was to analyze the dynamics of health insurance payments and expenditures for dental care in Bulgaria over the period 2019–2025, with a focus on differences between age groups and the balance between public funding and patient contributions. **Methods**: A retrospective descriptive–analytical study was conducted using aggregated data from NHIF annual reports, national legislation, and secondary literature. Absolute expenditures (BGN) and relative shares (%) of dental services within total health insurance payments were examined for the period 2019–2025 (actual data). Key dental procedures analyzed included examinations, restorations, extractions, and treatment of pulpitis/periodontitis in children, as well as prosthetic rehabilitation in older adults. Descriptive statistics, trend analysis, and simple linear regression were applied to assess expenditure growth and predictability. **Results**: Total health insurance payments in Bulgaria nearly doubled between 2019 and 2025, increasing from 4.12 to 8.87 million BGN. Dental expenditures rose from 167,000 to 416,000 BGN, with the share of dental care rising modestly from 4.05% to 4.69%. For children, NHIF covered nearly all costs, with minimal or absent co-payments. Among adults, a co-financing model prevailed, with fixed patient contributions for basic services but full NHIF coverage for prosthetic rehabilitation in edentulous patients aged 65–69 years. **Conclusions**: Bulgaria’s dental care financing reflects a socially oriented model, with full coverage for children and mixed financing for adults. Strategic policy adjustments are needed to ensure sustainability, equity, and alignment with universal health coverage goals.

## 1. Introduction

Oral health is a fundamental component of general health and well-being, yet oral diseases remain among the most widespread non-communicable conditions globally [1,2,3,4,5,6]. Dental caries, periodontal disease, and edentulism affect billions of people and contribute substantially to disability, pain, impaired daily functioning, and reduced productivity [1,2,6,7]. Despite being largely preventable, these conditions continue to impose significant social and economic burdens, particularly in lower-income and socially marginalized groups [3,4,8]. The WHO Global Oral Health Status Report calls for integrating oral health within universal health coverage (UHC) and restructuring financing models to support equitable access to essential dental services [3]. A consistent body of international evidence demonstrates that public financing arrangements play a decisive role in shaping access to dental care, utilization patterns, and the distribution of disease burden [4,9,10,11,12,13]. Countries with publicly funded or subsidized dental care exhibit lower socioeconomic inequalities in service use compared to systems heavily reliant on out-of-pocket payments [4,9,13]. Conversely, limited public financing correlates with higher rates of unmet need, treatment delay, and widening oral health disparities [8,14,15]. Recent analyses from Europe highlight that vulnerable groups—including older adults, low-income populations, and rural communities—face the strongest financial barriers to accessing timely dental care [12,16,17,18].

In Bulgaria, dental care is part of the mandatory health insurance system, regulated by the Health Insurance Act (promulgated SG, issue 70 of 1998, as amended). According to this law, all insured people are entitled to a defined range of dental services financed through the National Health Insurance Fund (NHIF). The financing and scope of dental care are governed by several key legislative and regulatory acts that determine access, types, and pricing of services. The main legal instruments include: (a) Health Insurance Act—regulates the principles of mandatory health insurance, the rights and obligations of insured persons, and the scope of medical and dental service packages financed by the NHIF [17]; (b) National Framework Contract (NFC) for Dental Services—concluded annually between the NHIF and the Bulgarian Dental Association (BDA), it specifies the dental activities included in the package for the given year, their costs, conditions for provision, and reporting requirements [19]; (c) Ordinance No. 3 of 2018 on the definition of the pack-age of health activities guaranteed by the NHIF budget—determines the specific volume of medical and dental services financed through the public health insurance fund [20]; (d) Health Act—establishes the general principles of public health protection, including prevention and health promotion, of which dental health is an integral part [16]; (e) Dental service package financed by the NHIF—differentiated by age groups, providing full or nearly full coverage for persons under 18 years of age, and a limited package for persons over 18. For children, the focus is on preventive check-ups, treatment of caries, pulpitis, and extractions. For adults, the package includes a limited number of services, with some interventions requiring co-payment by the patient. For patients over 65 years, specific prosthetic procedures are fully covered by the NHIF [21].

This design reflects features of a socially oriented system, consistent with European models prioritizing pediatric dental care and early preventive interventions [9,11,12]. However, structural challenges remain. Bulgaria is among the EU countries with higher household out-of-pocket dental spending, creating financial barriers for adults and older individuals [6,14,15]. Studies have documented persistent inequalities associated with income, education, geographic region, and demographic factors [14,15]. Broader health system pressures—including inflation, demographic aging, workforce shortages, and rising service costs—exert additional strain on the NHIF budget and influence access to dental care [12,18,22]. These issues mirror broader European trends, where increasing costs and shifting population needs require reforms in service coverage and financing strategies [10,13,14,22]. International policy frameworks emphasize that strengthening preventive services, adjusting benefit packages to better protect vulnerable populations, and periodically updating reimbursement tariffs are essential for enhancing equity and long-term sustainability in oral healthcare systems [3,4,5,10,22,23]. Yet empirical evidence assessing recent financing trends within Bulgaria’s NHIF—particularly during the post-2020 period characterized by inflationary pressures and service reorganization—remains limited.

The present study aimed to analyze the dynamics of health insurance payments and expenditures for dental care in Bulgaria during the period 2019–2025, with a focus on differences across age groups and payer types. The results could inform future policies directed at increasing the efficiency and equity of the dental health system.

## 2. Materials and Methods

### 2.1. Study Design and Scope

This study applied a retrospective descriptive–analytical design to examine the dynamics of health insurance payments and expenditures for dental care in Bulgaria over the period 2019–2025. The analysis focused on both children (under 18 years) and adults (18 years and above), with particular emphasis on differences in the structure of financing between the NHIF and patient co-payments.

### 2.2. Data Sources

The primary data were obtained from publicly available annual reports and official statistical documents of the NHIF, as well as relevant national legislation and regulatory frameworks governing dental healthcare financing (e.g., the Health Insurance Act, National Framework Contracts for Dental Services, Ordinance No. 3/2018).

The study relied exclusively on aggregated, publicly available annual data for the years 2019–2025, covering

- Total annual NHIF health expenditures.
- Total annual NHIF dental expenditures.
- NHIF and patient payments for five key dental procedures in children.
- NHIF and patient payments for the same procedures (plus prosthetic rehabilitation) in adults.

This resulted in 7 annual observations per indicator (2019–2025), consistent with the retrospective descriptive–analytical design of the study.

### 2.3. Operational Definitions of Dental Care Services

For the purpose of this study, dental care services were defined according to the terminology and procedural descriptions outlined in the NFC for Dental Services and the reporting standards of the National Health Insurance Fund (NHIF). The following service categories were included in the analysis:

1. Comprehensive oral examination with oral status assessment

A full diagnostic clinical evaluation that includes inspection of the oral cavity, assessment of dental and periodontal status, identification of treatment needs, and preparation of an outpatient dental record. This procedure represents the standard entry point into NHIF-financed dental care.

2. Restorations with dental (resin-based) composites

Placement of tooth-coloured composite restorative materials for the treatment of dental caries or for replacement of existing defective restorations. This category includes cavity preparation, restoration placement, finishing, and occlusal adjustment.

3. Extraction of a permanent tooth

Surgical removal of an erupted permanent tooth due to caries, trauma, periodontal disease, or other clinical indications. The procedure includes local anaesthesia, extraction, management of complications where applicable, and postoperative instructions.

4. Extraction of a primary tooth

Removal of a deciduous tooth when clinically indicated (e.g., carious destruction, infection, mobility, or interference with eruption of permanent teeth). The service includes local anaesthesia and standard postoperative care.

5. Treatment of pulpitis or periodontitis in a primary tooth

Endodontic treatment of primary teeth, including procedures such as pulpotomy or pulpectomy, removal of inflamed or necrotic pulp tissue, canal disinfection where applicable, and placement of a definitive restoration or sealing material.

6. Prosthetic rehabilitation for complete edentulism (upper or lower jaw)

Fabrication and delivery of complete dentures for patients aged 65–69 years with full edentulism of the upper or lower jaw. This category includes initial clinical assessment, impression taking, try-in sessions, delivery of the final prosthesis, and all follow-up examinations over a four-year period, as specified in the NFC.

### 2.4. Variables and Indicators

The study considered both absolute expenditures (in BGN) and relative shares (%) of dental services within total health insurance payments. The key indicators analyzed in this study included (1) NHIF expenditures for dental care, disaggregated by service category and year; (2) patient co-payments, recorded as fixed or variable amounts depending on the intervention and year; and (3) the share of dental care expenditures relative to total annual NHIF health insurance spending. For pediatric services, five core dental procedures were examined: (a) comprehensive oral examination with oral status assessment, (b) restorations with amalgam or dental composite materials, (c) extraction of permanent teeth, (d) extraction of primary teeth, and (e) treatment of pulpitis or periodontitis in primary teeth. For adults, the same set of procedures was analyzed, with the addition of prosthetic rehabilitation for complete edentulism of the upper or lower jaw in individuals aged 65–69 years. All indicators and service categories included in the study are consistently reflected in the Results, Discussion, and Conclusion sections.

### 2.5. Analytical Methods

Data were processed and visualized using descriptive statistics and trend analysis. Absolute and relative changes over time were calculated to assess the dynamics of expenditures. To evaluate the predictability of expenditure trends, simple linear regression models were applied separately for total NHIF health expenditures and for dental care expenditures. Simple linear regression was selected because the primary objective of the trend analysis was descriptive—to examine the direction, magnitude, and predictability of expenditure changes over time using annual aggregated data. Visual inspection of the time series (2019–2025) showed a stable, almost perfectly monotonic increase in both total NHIF expenditures and dental care expenditures, with no inflection points or cyclical patterns. Preliminary diagnostics (scatterplots and residual patterns) indicated a strong linear relationship between time and expenditures, which justified the use of a simple linear model. Given the small number of time points (*n* = 7), more complex models such as polynomial regression, autoregressive models, or exponential smoothing were considered but deemed inappropriate for several reasons: (1) their assumptions could not be robustly tested with the available data; (2) they offer limited additional interpretive value in short, annual datasets without seasonal or non-linear behavior; and (3) the aim of the study was not forecasting future expenditures but assessing the stability and direction of observed trends. The strength of associations was assessed using the coefficient of determination (R^2^). Graphical representations were constructed to illustrate both absolute and relative expenditure trends. All statistical analyses and visualization (descriptive statistics, trend analysis, and simple linear regression) were performed using Microsoft Excel 365, which is suitable for processing aggregated annual expenditure data and generating the graphical outputs.

### 2.6. Ethical Considerations

The study was based entirely on aggregated and publicly available data; therefore, no ethical approval or informed consent was required.

## 3. Results

### 3.1. Analysis of the Dynamics of Health Insurance Payments and Expenditures for Dental Care in Bulgaria (2019–2025)

**Figure 1:**
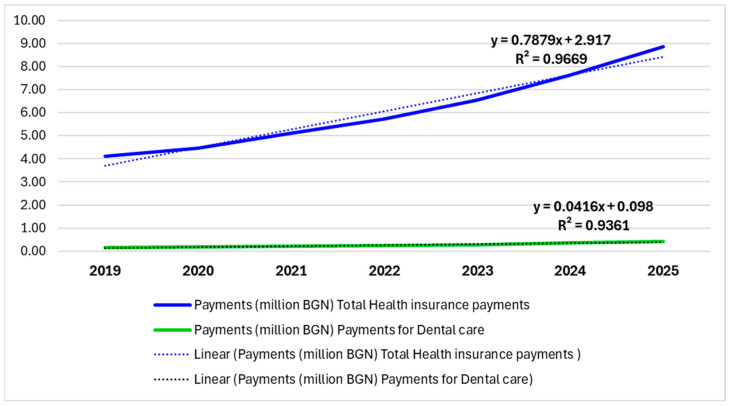
Trends in total health insurance payments and dental care expenditures in Bulgaria, 2019–2025.

**Figure 2:**
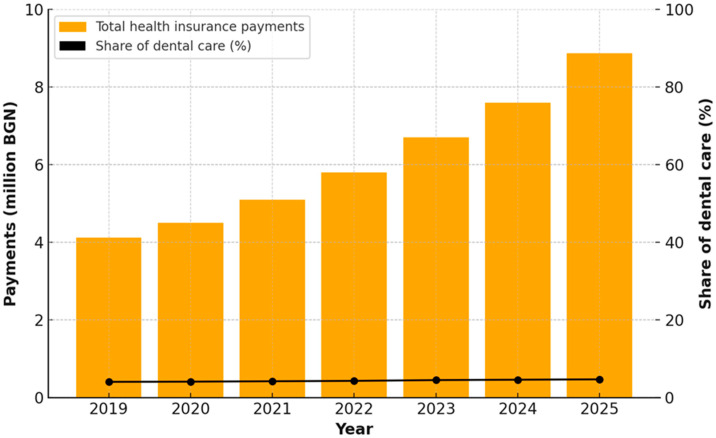
Absolute health insurance payments and proportion of dental care expenditures in Bulgaria, 2019–2025.

The present analysis examined the dynamics of health insurance payments in Bulgaria for the period 2019–2025, with particular emphasis on the position and development of dental care expenditures within the overall health insurance budget. Two complementary approaches to visualization and analysis were applied, presented in Figure 1 and Figure 2.

Figure 1 illustrates the absolute values of total health insurance payments and dental care expenditures, expressed in millions of BGN. The data analysis demonstrates a stable and clearly pronounced upward trend in total health insurance payments, which nearly doubled over the study period—from approximately 4.12 million BGN in 2019 to about 8.87 million BGN in 2025.

Dental care expenditures also increased over the same period—from around 167 thousand BGN in 2019 to approximately 416 thousand BGN in 2025.

To assess the predictability of these trends, an additional quantitative analysis was conducted using simple linear regression. The results revealed an exceptionally high degree of linear dependence for total health insurance expenditures, with the model explaining almost all of the variation in the data with R^2^ = 0.97 and R^2^ = 0.94, respectively. For dental care expenditures, a stable linear relationship was likewise identified, though with a lower slope of the regression line, reflecting a more moderate pace of growth.

Figure 2 provides a combined perspective on the absolute values of total health insurance payments and the relative share of dental care expenditures within them. In addition to the established upward trend in total expenditures, the figure reveals a slight increase in the relative share of dental expenditures—from about 4.05% in 2019 to approximately 4.69% in 2025.

### 3.2. Analysis of the Dynamics of Dental Services in Bulgaria, 2019–2025

#### 3.2.1. Analysis of the Dynamics of Dental Services Among Individuals Under 18 Years of Age in Bulgaria, 2019–2025

The present analysis traced the dynamics of key dental interventions among individuals under 18 years of age for the period 2019–2025, with emphasis on the structure of financing through the NHIF and patient co-payments. The study covers five major dental procedures and accounts for both the absolute values of expenditures and their changes over time.

- Comprehensive oral examination with oral status assessment and outpatient record preparation

**Figure 3:**
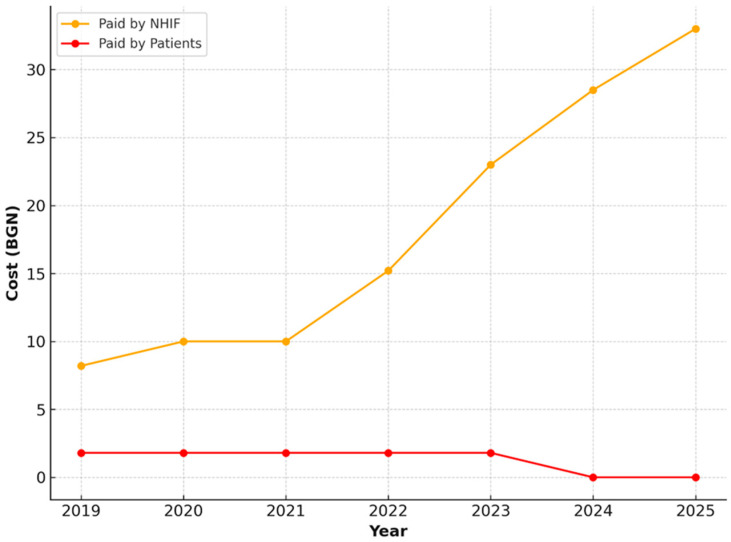
NHIF and patient payments for comprehensive oral examination for individuals under 18 years of age, 2019–2025.

For comprehensive examinations including oral status assessment and preparation of an outpatient record, a stable upward trend in NHIF-covered expenditures was observed. Values increase from 8.20 BGN in 2019 to 25.00 BGN in 2025. Patient payments remain significantly lower and practically unchanged (Figure 3).

- Restorations with Amalgam or Dental Composite

**Figure 4:**
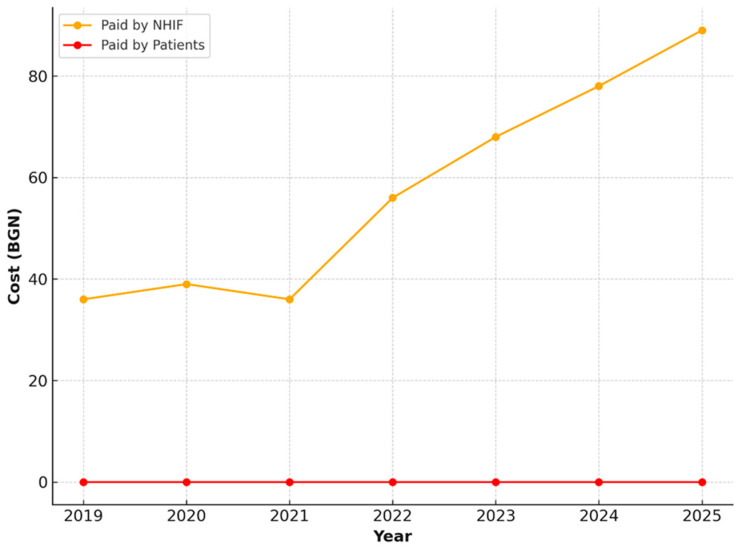
NHIF payments for restorations with amalgam or dental composite, 2019–2025.

Restorations with amalgam or dental composite demonstrated a similar trend. NHIF-covered expenditures increase steadily from 36.25 BGN in 2019 to 68.14 BGN in 2025. Throughout the entire period, patients do not incur any co-payments for this procedure, which underscores the priority given to tooth restoration in children within the publicly financed healthcare package (Figure 4).

- Extraction of a Permanent Tooth

**Figure 5:**
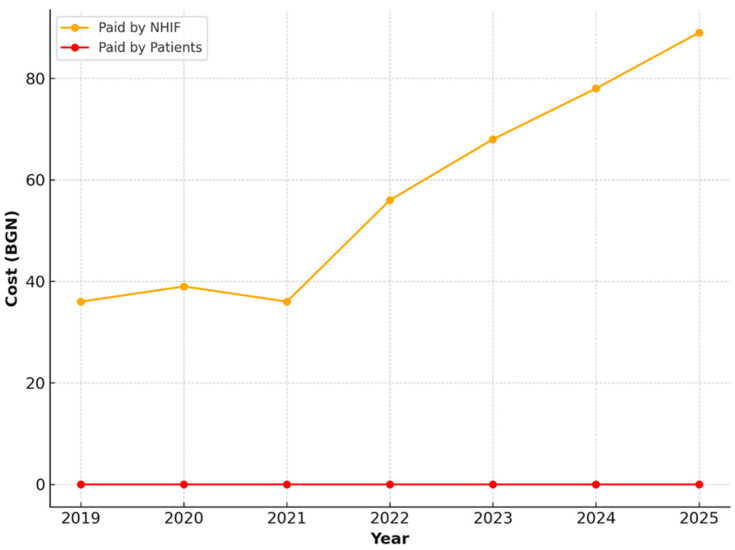
NHIF payments for extraction of a permanent tooth, 2019–2025.

For the extraction of a permanent tooth, including anesthesia, the expenditures increased from 36.25 BGN to 68.14 BGN during the study period. This intervention is also fully covered by the NHIF, without any financial contribution from patients (Figure 5).

- Extraction of a Primary Tooth

**Figure 6:**
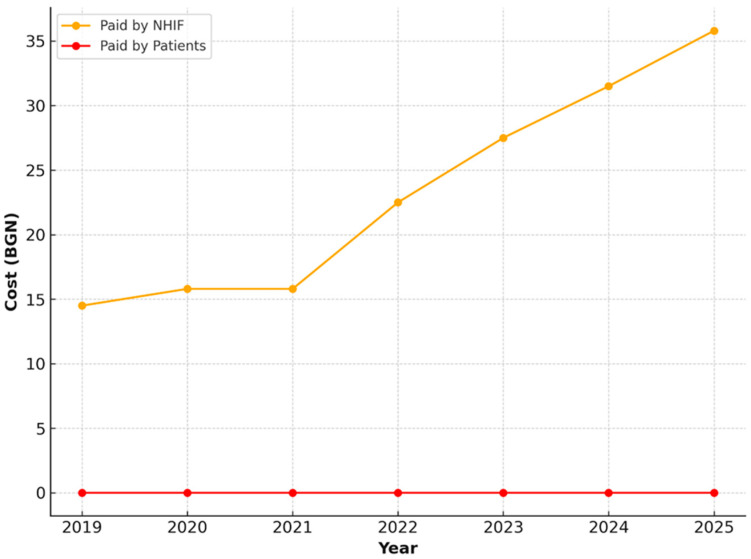
NHIF payments for extraction of a primary tooth, 2019–2025.

The extraction of a primary tooth showed a similar upward trend, though with lower absolute values ranging from 14.56 BGN to 27.38 BGN. In all years, financing was fully covered by the NHIF (Figure 6).

- Treatment of Pulpitis or Periodontitis in a Primary Tooth

**Figure 7:**
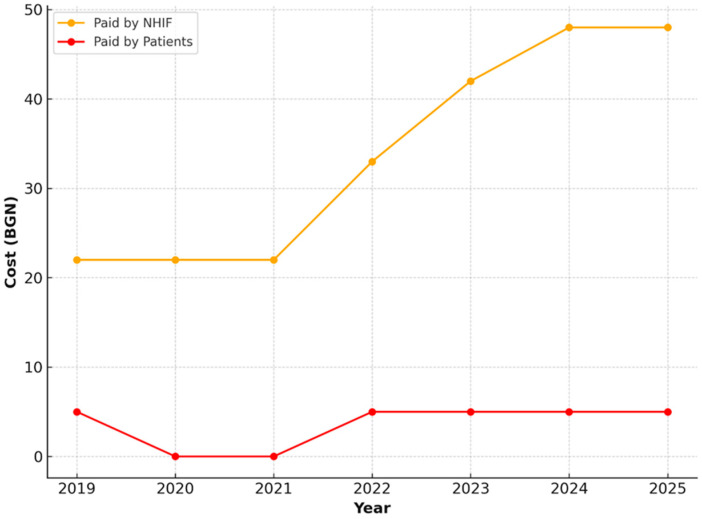
NHIF and patient payments for treatment of pulpitis or periodontitis in a primary tooth, 2019–2025.

The treatment of pulpitis or periodontitis in a primary tooth demonstrated a substantial increase in expenditure, rising from 26.62 BGN in 2019 to 46.30 BGN in 2025. An interesting aspect is that this procedure involved some degree of patient co-payment, which, although of low value, remains relatively constant over time (Figure 7).

In summary, the analysis of dental services among children under 18 years of age for the period 2019–2025 demonstrated a consistent trend of increasing expenditures and expanding public financing by the NHIF. In most cases, patients either did not make co-payments or contributed only symbolically, reflecting a high degree of social protection and equity in access to basic dental services. Exceptions were observed in certain more specific or therapeutic procedures, where partial patient payments existed, although with limited significance.

#### 3.2.2. Analysis of the Dynamics of Dental Services Among Adults in Bulgaria, 2019–2025

The analysis of key dental services for adults during the period 2019–2025 revealed clear trends both in terms of service volume and in the structure of financing between the NHIF and patients.

- Comprehensive oral examination with oral status assessment and preparation of an outpatient record

**Figure 8:**
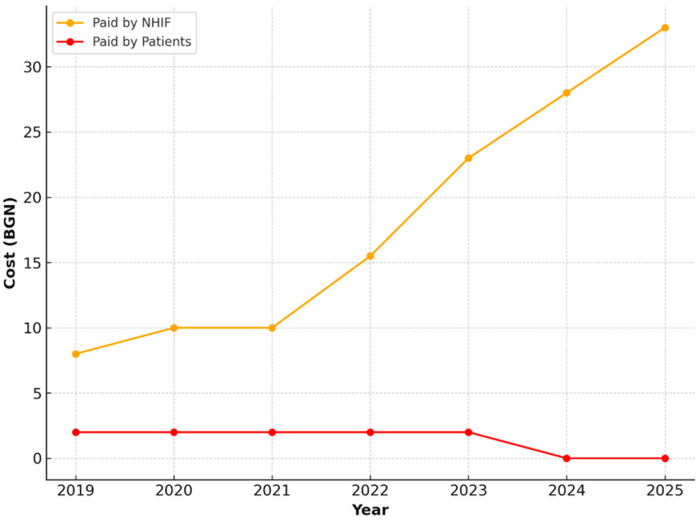
NHIF and patient payments for comprehensive oral examination for adult population, 2019–2025.

For comprehensive examinations including oral status assessment and preparation of an outpatient record, a steady increase in the NHIF-covered value of the service was observed. From 8.20 BGN in 2019, this value rises to 25.00 BGN in 2025. Patient payments were considerably lower—around 1.80 BGN—and remained unchanged over time (Figure 8).

- Restorations with Amalgam or Dental Composite

**Figure 9:**
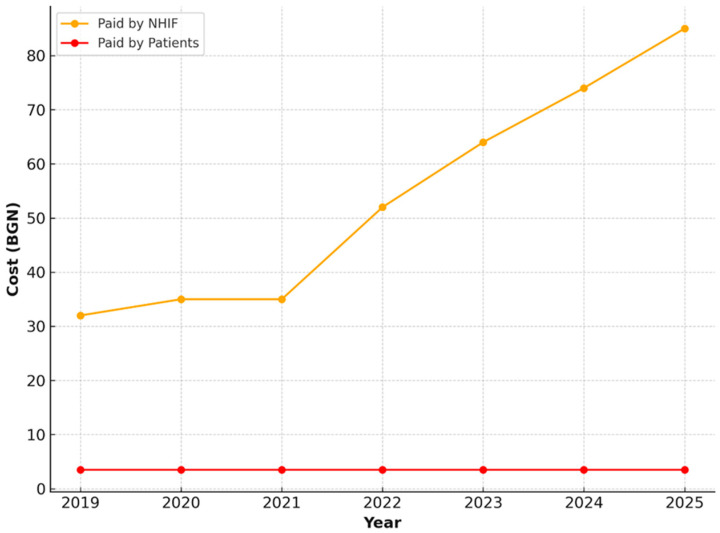
NHIF and patient payments for restorations with amalgam or dental composite, 2019–2025.

For restorations with amalgam or dental composite, an increase was also observed in the amount covered by the NHIF—from 32.25 BGN in 2019 to 68.14 BGN in 2025. Patients contributed a fixed co-payment of 4.00 BGN, which remains unchanged throughout the period (Figure 9).

- Extraction of a Permanent Tooth

**Figure 10:**
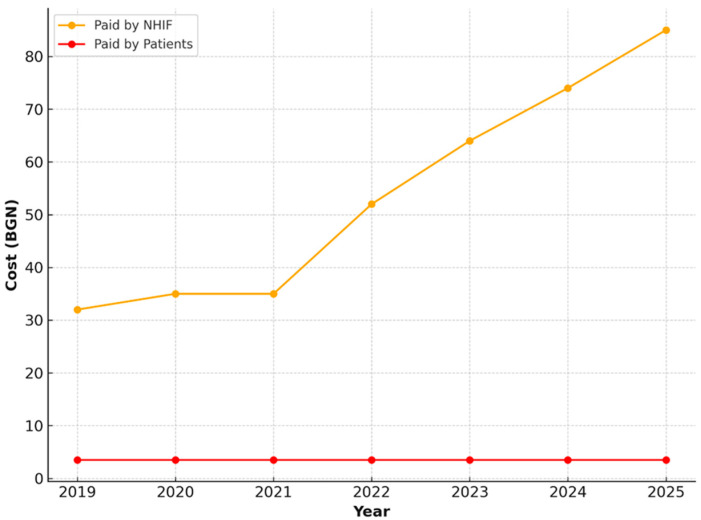
NHIF and patient payments for extraction of a permanent tooth, 2019–2025.

The extraction of a permanent tooth, including anesthesia, followed an identical pattern—the value covered by the NHIF increased progressively, while the patient co-payment remained unchanged (Figure 10).

Activities for Restoring the Function of the Masticatory Apparatus in Completely Edentulous Upper or Lower Jaws, Including Follow-Up Examinations over a 4-Year Period in Individuals Aged 65–69 Years

**Figure 11:**
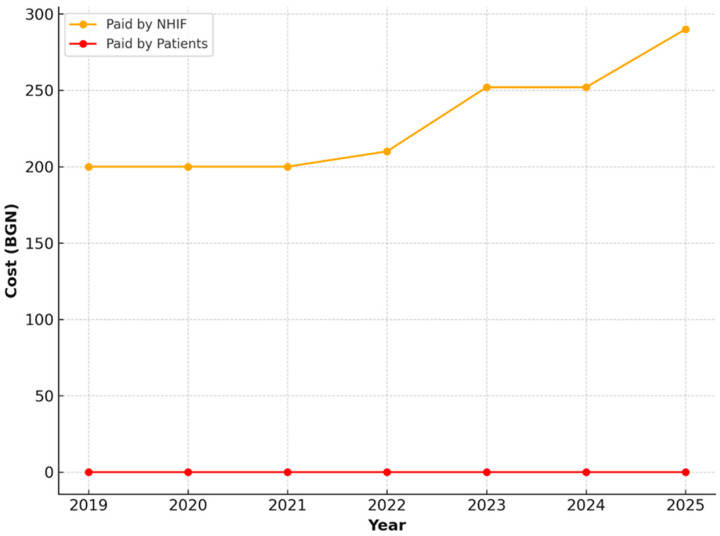
NHIF payments for restoration of masticatory function in a completely edentulous upper jaw, including follow-up examinations, in patients aged 65–69 years (2019–2025).

**Figure 12:**
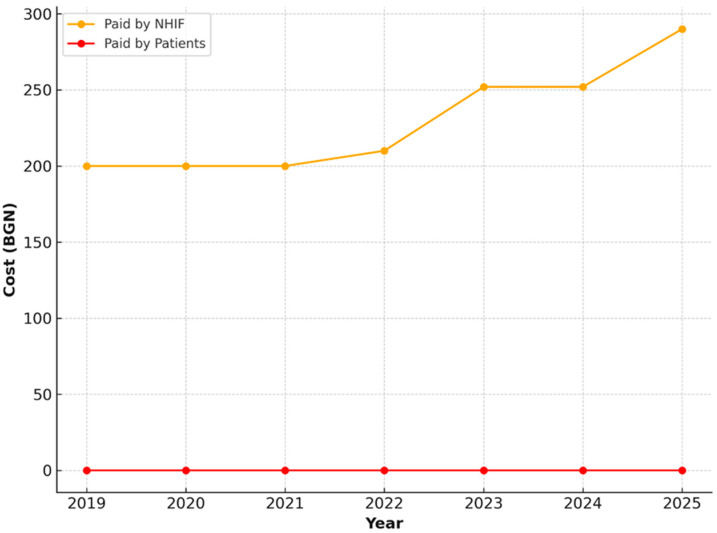
NHIF payments for restoration of masticatory function in a completely edentulous lower jaw, including follow-up examinations, in patients aged 65–69 years (2019–2025).

For activities related to the restoration of masticatory function in cases of complete edentulism—both upper and lower jaws—only public financing provided by the NHIF was observed. The value of these services remained stable over the years, consistently set at 200.00 BGN, without any patient co-payment (Figure 11 and Figure 12).

In summary, the data from the period 2019–2025 showed a consistent increase in the value of key dental services covered by the NHIF for patients over 18 years of age. Patient co-payments remained stable and relatively low or were entirely absent from certain services.

## 4. Discussion

This study provides a detailed examination of the dynamics of NHIF-funded dental care in Bulgaria from 2019 to 2025, revealing important trends in public financing, service utilization, and the broader structural factors influencing oral health care. These findings shed light on how Bulgaria’s dental system is evolving within the context of rising costs, demographic changes, and long-standing inequalities. Understanding these dynamics is essential for informing evidence-based policy reforms that strengthen equity, sustainability, and alignment with international best practices.

### 4.1. Dental Care Financing in Bulgaria in the European Context

Oral health care in Bulgaria, as in many European countries, is historically separated from general health care provision. Public funding covers only a portion of total dental costs, and significant variability persists in access, utilization, and financial protection [9,10,18]. This partial integration contributes to enduring inequalities, as household income remains a major determinant of whether individuals can afford preventive and restorative dental care. Despite operating a mandatory health insurance system, Bulgaria consistently reports high levels of out-of-pocket spending on dental services, similar to trends observed across Central and Eastern Europe (CEE) [6,14,24,25].

These structural patterns are consistent with international findings showing that countries with limited public dental coverage exhibit greater social inequalities in dental service use and worse oral health outcomes [4,9,13]. By contrast, systems with broader public financing—such as those in Nordic countries—report lower unmet needs and more equitable service distribution [11,12,26].

### 4.2. Interpretation of Financing Dynamics, 2019–2025

The results of this study show a clear and consistent increase in NHIF payments for dental services between 2019 and 2025. This trend aligns with global and European movements to strengthen oral health’s role within universal health coverage frameworks [1,2,3,5,8]. The upward shift likely reflects multiple factors: inflation, increased service demand, updated reimbursement tariffs, and the need to align NHIF payments with the real costs incurred by dental practices.

International research demonstrates similar patterns in many countries, where increases in dental material prices, infection control requirements, and workforce costs have prompted periodic adjustments to reimbursement levels to maintain provider participation [7]. Bulgaria’s rising NHIF financing mirrors this broader European trend.

Despite these positive developments, the share of dental spending relative to overall NHIF expenditure increased only modestly, from approximately 4.0% to 4.7%. This finding confirms that dentistry remains a relatively small—and often underfunded—component of Bulgarian health care spending, similar to other EU systems where oral health remains peripheral to major health financing decisions [11,13,22].

### 4.3. Public Financing for Children: A Strong Socially Oriented Model

One of the most notable findings is the consistently high level of public financing for pediatric dental services. For individuals under 18, all major interventions—including examinations, restorations, and extractions—are almost entirely financed by the NHIF, with minimal co-payments limited to specific pulp treatments. This approach is strongly aligned with WHO guidance emphasizing early-life prevention, routine examinations, and full coverage of children’s dental services as essential components of a modern public health strategy [3].

Comparable systems across Europe, such as those in Finland and Sweden, offer free or heavily subsidized dental care for children and adolescents and have demonstrated long-term reductions in disease burden, inequalities, and financial barriers [11]. Bulgaria’s model therefore reflects global best practices and constitutes a significant strength of the national oral health care system [27].

### 4.4. Adult Coverage: Persistent Co-Payments and Implications for Equity

For adults, the study identifies a mixed public–private financing model. NHIF covers a substantial proportion of essential services; however, co-payments remain necessary for examinations, restorations, and extractions. While these co-payments are modest, international evidence shows that even small out-of-pocket charges can reduce the likelihood of seeking treatment, particularly among low-income and unemployed individuals [4,15].

This pattern is typical in many CEE countries, where adult dental care is partially subsidized but still financially burdensome for vulnerable populations [12,28]. Germany uses a similar co-payment system, although supplementary health insurance often mitigates financial burden—an option less available in Bulgaria [4]. Thus, although Bulgaria shows a relatively high share of public financing, adults remain at greater risk of unmet needs compared to children.

A noteworthy positive exception is the full public coverage of prosthetic rehabilitation for adults aged 65–69. Given the high prevalence of edentulism and masticatory dysfunction in this age group, such support is critical. This puts Bulgaria in a more favorable position compared to countries like the United Kingdom, where prosthetic services often require substantial private payments or are predominantly delivered in the private sector [7].

### 4.5. Structural Drivers of Observed Trends

#### 4.5.1. Policy Environment

Bulgaria’s health financing policies—such as external reference pricing, HTA procedures, and annual NHIF negotiations—have aimed to manage costs and maintain system sustainability. However, despite these mechanisms, expenditures on outpatient and pharmaceutical services continue to rise [6]. The limited publicly funded preventive dental services—particularly for adults—contribute to high unmet needs and persistently high rates of restorative and extraction procedures [13,25]. Evidence from other nations shows that legal mandates for preventive dental coverage lead to better oral health outcomes, particularly among children [13].

#### 4.5.2. Demographic Shifts

Population aging, urban–rural disparities, and socioeconomic inequalities significantly shape dental service utilization. Elderly individuals—especially in rural areas—report higher unmet needs due to financial and transportation barriers [12,25,29]. Workforce distribution challenges, such as dentist concentration in urban centers and the rise of corporate dental chains, may further limit availability in underserved regions [29]. Educational attainment, employment, and social support are also strong predictors of dental service use, with disadvantaged groups utilizing fewer services [25].

#### 4.5.3. Economic Barriers

Out-of-pocket payments remain a central barrier to care in Bulgaria [6,25]. Economic growth has not kept pace with rising health expenditures, and dental care is disproportionately forgone by low-income groups [12]. This aligns with international findings showing that oral conditions impose a high economic burden due to direct treatment costs and productivity loss [30]. Inflationary pressures also affect the ability of dental practices to deliver services under static reimbursement levels, reinforcing the need for periodic tariff adjustments [22].

### 4.6. Overall Synthesis

The interplay of limited public financing for adults, high out-of-pocket spending, demographic inequalities, and evolving dental practice models underpins the observed trends in Bulgaria’s dental service landscape from 2019 to 2025. Although public financing has increased, particularly for pediatric care and prosthetic services for older adults, significant inequalities persist. Co-payments for adults remain a barrier to equitable access, and preventive services remain underfunded relative to restorative treatments.

### 4.7. Implications for Policy and Future Reform

1. To improve oral health outcomes in Bulgaria, reforms should focus on.
2. Expanding publicly funded preventive care, especially for adults, to reduce long-term disease burden.
3. Adjusting co-payment structures, potentially using income-based models to protect vulnerable groups.
4. Enhancing regional and socioeconomic equity through targeted programs for rural, elderly, and low-income populations.
5. Increasing public expenditure on adult dental care to align with evidence showing that stronger public financing improves outcomes [13,23].
6. Strengthening data collection on utilization, unmet needs, and inequalities to support evidence-based policy decisions.
7. Regularly updating reimbursement tariffs to reflect increased practice costs and ensure provider sustainability.

### 4.8. Limitations

This study has several limitations that should be acknowledged. First, the analysis is based on aggregated data on health insurance payments and expenditures for dental services, which does not allow for assessment at the level of individual patients or dental practices. As such, important variations related to socioeconomic status, geographic distribution, or specific patient groups could not be captured. Second, the study focuses primarily on the financial dimension of dental care and does not assess clinical outcomes or oral health status indicators, which would provide a more comprehensive picture of the effectiveness of financing models. Third, while the period 2019–2025 offers valuable insights into recent trends, it overlaps with the COVID-19 pandemic, which may have introduced temporary distortions in service utilization and funding patterns. Fourth, international comparisons were drawn primarily from published literature, which may differ in methodology, definitions of service coverage, or reporting standards, limiting the direct comparability of findings. Finally, the study does not account for potential future policy changes, inflationary shocks, or demographic shifts beyond 2025, which may significantly influence the sustainability of dental healthcare financing in Bulgaria.

## 5. Conclusions

This study analyzed the dynamics of health insurance payments and dental care expenditures within the Bulgarian healthcare system between 2019 and 2025. The findings show a clear upward trend in both total NHIF spending and publicly financed dental services, with dental expenditure increasing in absolute terms and maintaining a relatively stable share of the overall budget. For children, the financing model remains strongly socially oriented, with almost complete public coverage and minimal co-payments. For adults, a mixed model persists, characterized by fixed co-payments for basic procedures and full NHIF coverage for selected services such as prosthetic rehabilitation among older adults.

These patterns highlight three important conclusions. First, public financing for dental care in Bulgaria has expanded in recent years, reflecting efforts to maintain access despite economic pressures and increasing service costs. Second, the structure of financing differs markedly by age group, indicating that the system provides strong protection for children but only partial protection for adults, which may contribute to persistent inequalities in oral health. Although the present study did not directly measure or visualize health inequalities the different financing structures by age group may have implications for equity, particularly for vulnerable adults facing fixed co-payments. Third, the observed trends underscore the need for continued policy attention to the balance between public coverage, patient contributions, and long-term financial sustainability.

In this context, strategic planning and adjustments to the health insurance package are needed. The observed trends—rising costs, expansion of publicly funded services, and stable but potentially burdensome co-payments for adults—show that the current socially oriented model remains strong but may require refinement to meet future challenges. Ensuring that financial protection keeps pace with service costs, demographic changes, and utilization patterns will be essential to safeguard both equity and sustainability.

Based on these insights, future policy development should focus on (1) expanding preventive coverage, (2) adjusting adult co-payments based on income, (3) extending full NHIF financing to vulnerable adult groups, (4), using the National Framework Contract to promote equitable access, (5) improving national monitoring infrastructures, and (6) revising reimbursement tariffs regularly. Such measures would support a more equitable and sustainable dental financing model while helping to improve the oral health of the Bulgarian population.

## Data Availability

The data supporting the findings of this study are publicly available from official sources of the NHIF of Bulgaria and the Ministry of Health. These include annual NHIF reports (https://tinyurl.com/bdk3x7y6 (accessed on 2 October 2025)) and national legislative documents regulating dental healthcare financing (e.g., the Health Insurance Act—https://www.minfin.bg/upload/36670/Health_Insurance_Act.pdf (accessed on 2 October 2025), National Framework Contracts for Dental Services—https://tinyurl.com/477rtkzw (accessed on 2 October 2025), and Ordinance No. 3/2018—https://www.mh.government.bg/upload/4196/naredba_3-2018.pdf (accessed on 2 October 2025)). All data used in the analysis were aggregated and retrieved from publicly accessible repositories. No new data was created or collected specifically for this study. No restricted, confidential, or proprietary sources were used, and all data can be freely consulted and verified by any reader.

## Abbreviations

The following abbreviations are used in this manuscript:

BDA	Bulgarian Dental Association
BGN	Bulgarian lev
NFC	National Framework Contract
NHIF	National Health Insurance Fund
SG	State Gazette
